# Effects of Process Parameters on Tensile Properties of 3D-Printed PLA Parts Fabricated with the FDM Method

**DOI:** 10.3390/polym17141934

**Published:** 2025-07-14

**Authors:** Seçil Ekşi, Cetin Karakaya

**Affiliations:** 1 Mechanical Engineering Department, Sakarya University, Sakarya 54100, Turkey; 2 Independent Researcher, Hastings, East Sussex TN34 1EN, UK; cetkarakaya@gmail.com

**Keywords:** fused deposition modeling, printing parameters, PLA, tensile strength, optimization

## Abstract

This study investigates the influence of key fused deposition modeling (FDM) process parameters, namely, print speed, infill percentage, layer thickness, and layer width, on the tensile properties of PLA specimens produced using 3D printing technology. A Taguchi L9 orthogonal array was employed to design the experiments efficiently, enabling the systematic evaluation of parameter effects with fewer tests. Tensile strength and elongation at break were measured for each parameter combination, and statistical analyses, including the signal-to-noise (S/N) ratio and analysis of variance (ANOVA), were conducted to identify the most significant factors. The results showed that infill percentage significantly affected tensile strength, while layer thickness was the dominant factor influencing elongation. The highest tensile strength (47.84 MPa) was achieved with the parameter combination of 600 mm/s print speed, 100% infill percentage, 0.4 mm layer thickness, and 0.4 mm layer width. A linear regression model was developed to predict tensile strength with an R^2^ value of 83.14%, and probability plots confirmed the normal distribution of the experimental data. This study provides practical insights into optimizing FDM process parameters to enhance the mechanical performance of PLA components, supporting their use in structural and functional applications.

## 1. Introduction

In recent years, additive manufacturing (AM) has emerged as a transformative technology that is reshaping various industries by enabling rapid, flexible, and cost-effective production methods. Unlike traditional subtractive or formative manufacturing processes, AM builds objects layer by layer directly from digital models, allowing for complex geometries, material efficiency, and mass customization [1,2,3,4,5]. Among the various AM techniques, fused deposition modeling (FDM) is one of the most prevalent due to its affordability, ease of operation, and wide material availability. FDM extrudes a thermoplastic filament through a heated nozzle, depositing material in successive layers to create the desired three-dimensional geometry [6,7].

One of the most commonly used materials in FDM is polylactic acid (PLA), a biodegradable, bio-based polymer derived from renewable resources such as corn starch or sugarcane [8,9]. PLA is favored for its low melting temperature, minimal warping, and environmental friendliness, making it suitable for prototyping, biomedical devices, packaging, and educational tools [10,11]. However, the mechanical performance of PLA parts produced by FDM is often inferior to that of traditional methods due to layer interfaces, voids, and poor interlayer adhesion. The printing process parameters largely influence this performance variability, directly affecting the printed layers’ quality, density, and bonding [12,13].

Numerous studies have demonstrated that process parameters such as layer thickness, infill density, print speed, nozzle diameter, build orientation, and temperature profoundly impact the mechanical properties of FDM-printed parts [14,15]. For example, a lower layer height can improve the interlayer bonding and surface finish, enhancing tensile strength [16,17]. Similarly, increasing the infill density improves the internal structure and reduces porosity, positively affecting tensile, flexural, and impact resistance. However, this comes at the expense of increased material usage and longer printing times, necessitating an optimization trade-off [18,19,20,21].

Print speed and layer width influence the extrusion rate, cooling behavior, and layer fusion, all critical for mechanical integrity. Faster print speeds may reduce build time but can lead to weak bonding and defects such as delamination or under-extrusion. On the other hand, the nozzle diameter determines the width of the extruded filament and affects both resolution and mechanical interlocking between layers. Furthermore, the temperature settings for the nozzle and print bed can impact the degree of crystallinity and residual stresses within the printed part, thereby affecting stiffness and ductility [22,23].

Researchers have employed various experimental design and analysis techniques to identify the optimal process settings. The Taguchi method, in particular, offers a systematic approach to minimize the number of experiments required to study multiple factors at different levels [24,25]. Combined with a signal-to-noise (S/N) ratio analysis, it provides insight into the robustness and sensitivity of the system under investigation. Moreover, analysis of variance (ANOVA) is widely used to assess each parameter’s statistical significance and percentage contribution to the output responses [26,27]. In recent years, predictive modeling approaches such as linear regression, artificial neural networks, and support vector machines have been employed to estimate mechanical properties based on input parameters, further supporting process optimization in FDM [28,29,30].

While much research has been devoted to analyzing tensile properties, the mechanical behavior of PLA parts under impact, flexural, and fatigue loading conditions is equally critical for structural and functional applications [6,31]. For instance, Milovanović et al. [32] reported that the layer thickness significantly affects FDM-printed PLA’s impact strength and energy absorption capability. Similarly, research by Gupta et al. and Rajpurohit et al. [33,34] demonstrated that the raster angle influences flexural and tensile properties.

Fatigue resistance, an essential factor in load-bearing applications, has been studied by several researchers. Javadian et al. [35] investigated the fatigue behavior of 3D-printed continuous glass fiber-reinforced PLA composites. Moreover, Talati-Ahmad et al. [36] investigated metamaterial structure impacts on stress and bending fatigue lifetimes of additive-manufactured 3D-printed PLA specimens. Kianifar et al. studied the effect of simulated body fluid on the fatigue resistance of 3D-printed PLA and PLA–wood structures under cyclic bending loading. The results indicate that PLA–wood composites exhibit a superior fatigue lifespan compared to pure PLA. Immersion in SBF notably decreased the fatigue lifespan of both PLA and PLA–wood composites [37]. Kiani et al. investigated corrosion behavior, tensile properties, and high-cycle fatigue performance of 3D-printed PLA/20 wt%PCL blend specimens under non-corrosive and corrosive (SBF) conditions The results indicate that 3D-printing of PLA/PCL blends is a promising technique to achieve bioabsorbable components with improved mechanical properties in physiological environments, compared with pure PLA [38].

Despite the growing body of literature, integrated studies are still needed to systematically examine the combined effect of multiple FDM parameters on the mechanical behavior of PLA parts using structured design methods and statistical tools. Many studies tend to isolate one or two parameters, limiting their findings’ generalizability and practical utility. Moreover, few works employ probabilistic methods such as probability plots to verify the statistical assumptions and distributional characteristics of the data, which is important for the validity of subsequent analyses and modeling.

In this context, the present study aims to fill this gap by evaluating the influence of four critical FDM process parameters, printing speed, infill percentage, layer thickness, and layer width, on the tensile properties of PLA specimens. A Taguchi L9 orthogonal array is used for efficient experimental design, and ISO standards are used to conduct tensile tests to assess performance. An S/N ratio analysis determines the optimal parameter settings, while ANOVA is applied to identify statistically significant parameters. Furthermore, a linear regression model is developed to predict tensile strength based on the selected input variables, and probability plots are constructed to examine the distribution of the experimental data. The findings of this study are expected to contribute to the body of knowledge on FDM process optimization and provide practical guidelines for enhancing the mechanical performance of PLA-based components, particularly for applications requiring structural reliability and material efficiency.

## 2. Materials and Methods

**Figure 1:**
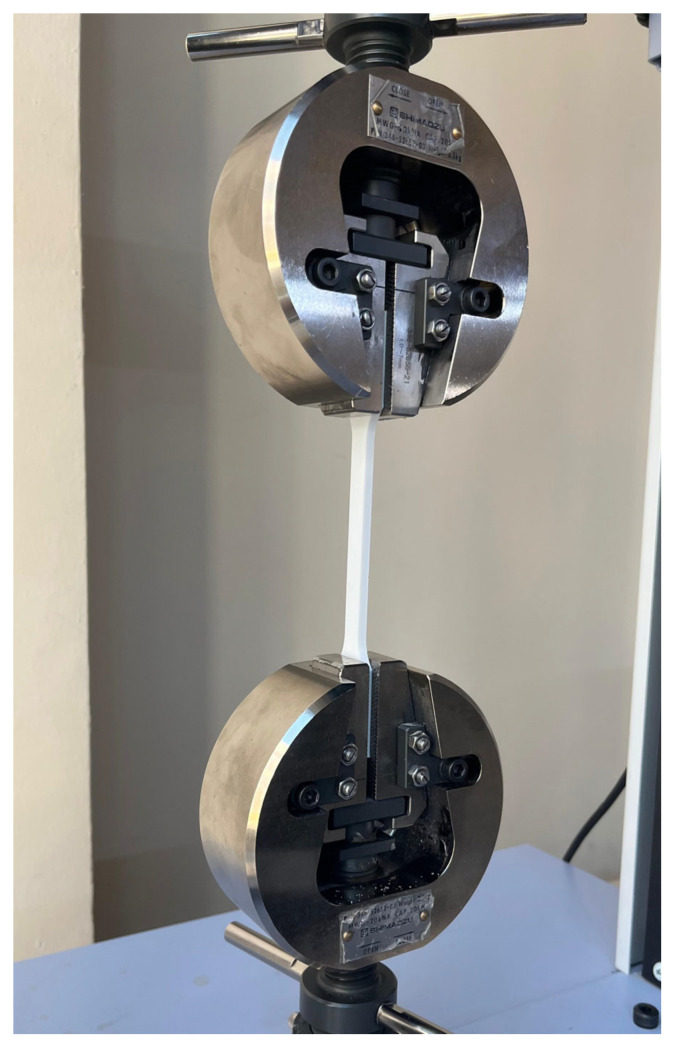
View of the test specimens during tensile tests.

**Figure 2:**
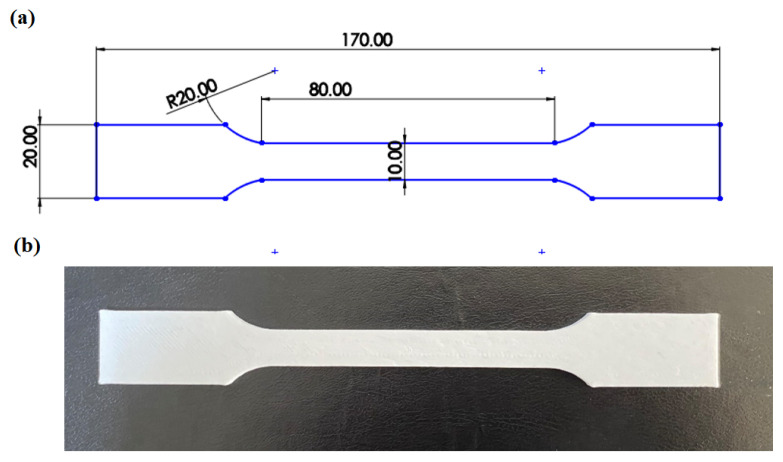
Test specimen: (**a**) ISO 527-2 Type B standard tensile test specimen; (**b**) 3D-printed PLA tensile test specimen.

In this research, tensile test specimens were fabricated from PLA material using the fused deposition modeling (FDM) technique while varying the process parameters on a 3D printer. The specimen geometry complied with the ISO 527-2 standard. PLA, a thermoplastic polymer derived from renewable sources such as corn and sweet potatoes, is known for its biodegradability. It is commonly utilized in applications ranging from additive manufacturing to food packaging and medical devices. A Creality K1 3D printer (Shenzhen, China) was employed for the fabrication process. The primary process parameters in the experiments included print speed, infill percentage, layer thickness, and layer width. The Creality Print 5.0 software was used as the slicer. A rectilinear infill pattern was used in this study. Nozzle and bed temperatures were maintained constant across all trials. Tensile testing was performed using a Shimadzu universal testing machine with a 10 kN load capacity at a constant crosshead speed of 2 mm/min. A view of the test specimens during the tensile tests is given in Figure 1. A view of the tensile test specimen is given in Figure 2. The experiments were repeated 3 times. The experiments were carried out at room temperature. Humidity was kept constant.

In this section, statistical evaluations of the tensile test results are carried out using the Minitab 15 software with a 95% confidence interval. The experimental setup was structured based on the Taguchi design methodology, which was utilized to determine the most effective combination of process parameters while minimizing the number of test runs. Specifically, an L9 (3^2^) orthogonal array was employed to efficiently assess the influence of four key FDM parameters, print speed, infill percentage, layer thickness, and layer width, each tested at three distinct levels. 

The mechanical behavior of the PLA specimens was investigated through uniaxial tensile tests. The outcomes were analyzed using the signal-to-noise (S/N) ratios to identify the optimal parameter levels and assess the consistency of the results. Analysis of variance (ANOVA) was also applied to determine each parameter’s statistical significance and relative impact on tensile strength and elongation at break. Among the three standard S/N evaluation criteria, “smaller is better”, “nominal is best”, and “larger is better”, the “larger is better” approach was chosen, in alignment with the study’s objective of maximizing tensile performance. The S/N ratio was computed using Equation (1), where *y_i_* denotes the measured response and *n* indicates the number of observations.

(1) $$S/N\;=-10log\left(\frac{1}{n}\sum_{i=1}^{n}\frac{1}{y_{i}^{2}}\right)$$

The process parameters and their corresponding level values are given in Table 1. The parameters and levels were chosen according to the print limitations of the 3D printer. The L9 orthogonal array for the design of experiments is given in Table 2.

Analysis of variance (ANOVA) was performed to analyze the effect of the parameters on tensile strength.

## 3. Results and Discussion

**Figure 3:**
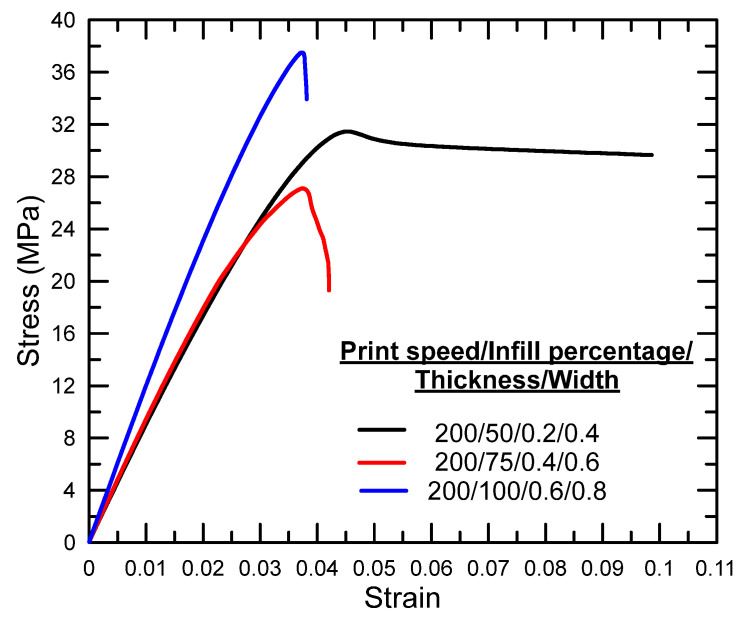
Stress–strain graphs for experiments 1–3.

**Figure 4:**
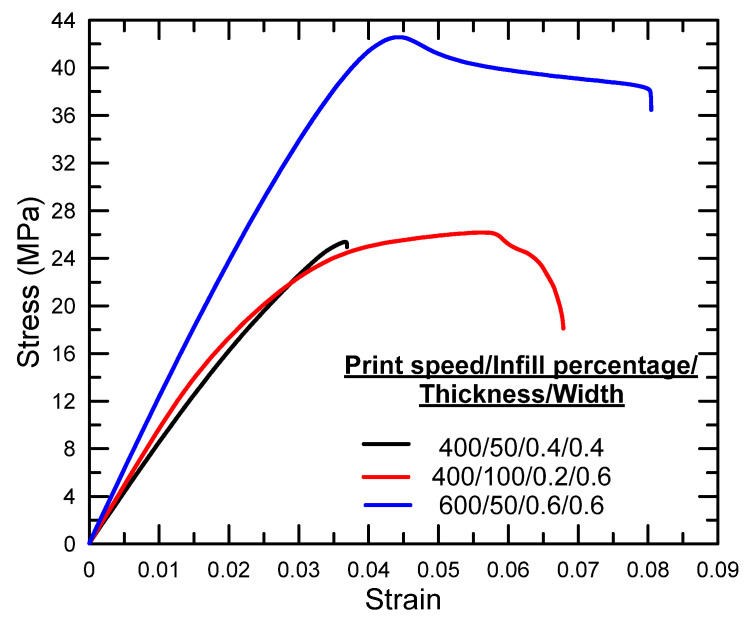
Stress–strain graphs for experiments 4–6.

**Figure 5:**
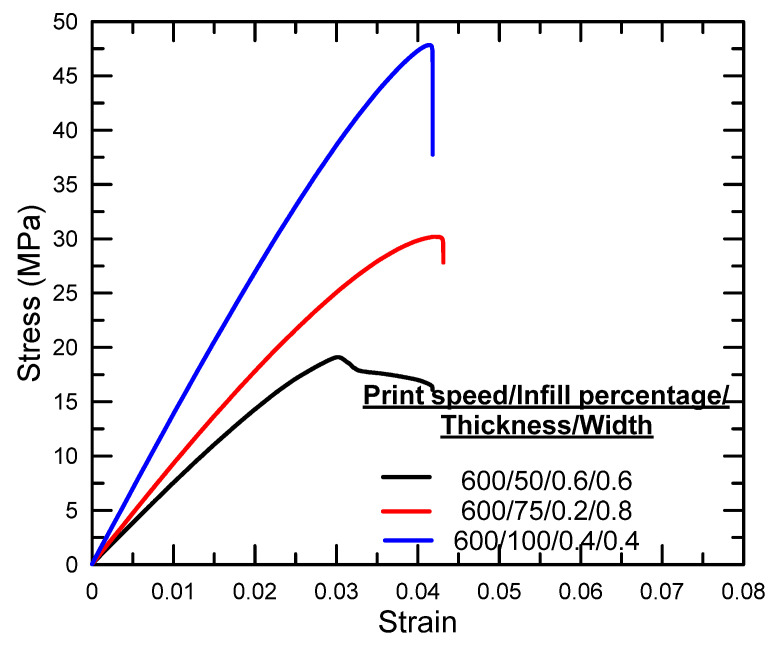
Stress–strain graphs for experiments 7–9.

Figure 3, Figure 4 and Figure 5 illustrate the tensile stress–strain curves obtained for each specimen fabricated using the Taguchi L9 orthogonal array. The corresponding tensile strength, percentage elongation values, and the signal-to-noise (S/N) ratio results derived from the Taguchi analysis are summarized in Table 3. Specimen 9 exhibited the highest ultimate tensile strength among the tested configurations, whereas specimen 7 demonstrated the lowest. In terms of ductility, the greatest elongation at break was recorded for specimen 1, while specimen 4 showed the lowest elongation.

**Figure 6:**
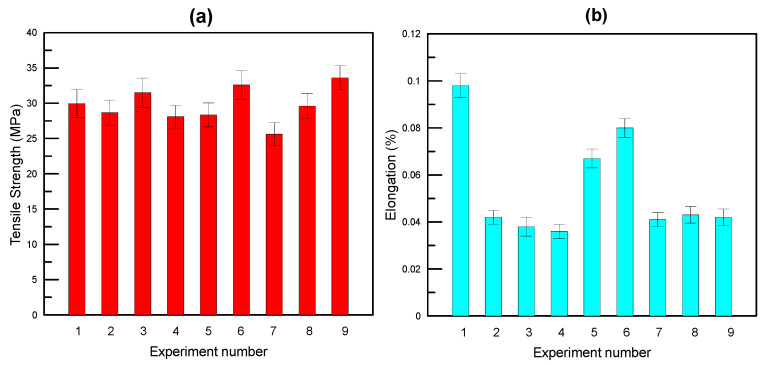
Tensile test results. (**a**) Tensile strength; (**b**) % elongation.

Results obtained from tensile tests are given in Figure 6.

Table 4 presents the response values for the S/N ratios corresponding to tensile strength and the ranking of parameter significance based on their impact. Among the examined variables, infill percentage emerged as the most influential factor affecting tensile strength, followed by layer thickness, layer width, and finally print speed, which had the least effect. It has also been emphasized in similar studies that filling percentage and layer thickness are important factors [10,28]. Similarly, Table 5 summarizes the S/N ratio responses for percentage elongation. In this case, layer thickness had the most significant influence, followed by layer width and print speed, while infill percentage exhibited the lowest effect on elongation behavior.

**Figure 7:**
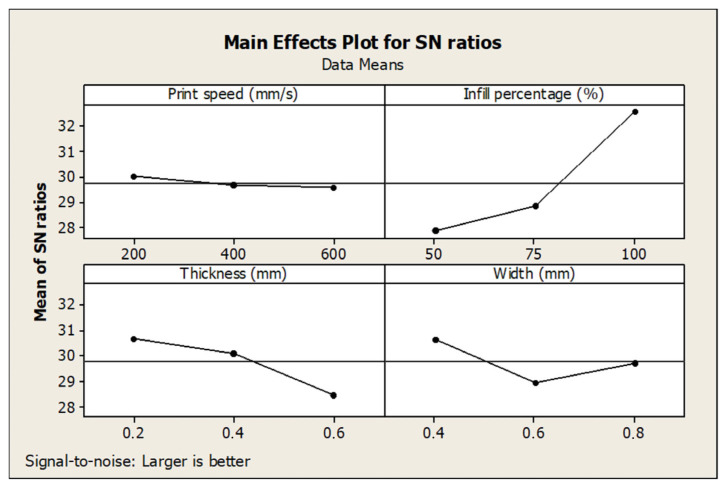
Main effect plot of S/N for the tensile strength.

**Figure 8:**
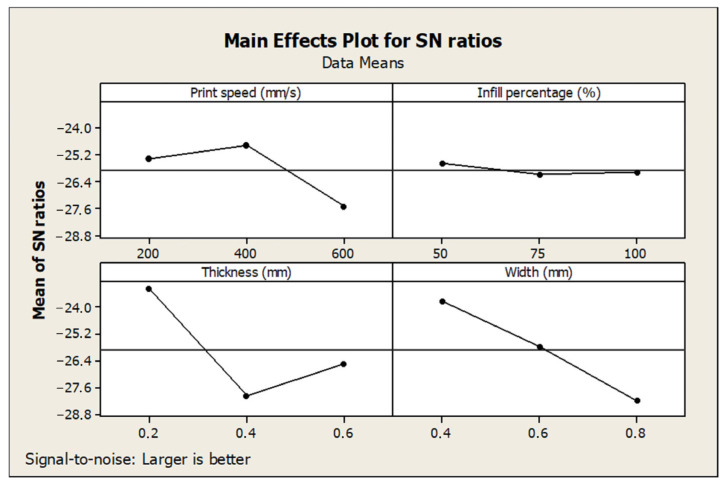
Main effect plot of S/N for the % elongation.

The main effect plots for the S/N ratios related to tensile strength and elongation are illustrated in Figure 7 and Figure 8, respectively. According to the analysis, the optimal combination of parameters for achieving the highest tensile strength was identified as 600 mm/s print speed, 100% infill percentage, 0.4 mm layer thickness, and 0.4 mm layer width (A3B3C2D1), which resulted in a maximum tensile strength of 47.84 MPa. Conversely, the parameter combination yielding the greatest elongation was determined as 200 mm/s print speed, 50% infill percentage, 0.2 mm layer thickness, and 0.4 mm layer width (A1B1C1D1). These findings demonstrate the trade-off between stiffness and ductility in FDM printing and highlight the need for parameter tuning based on specific application requirements.

As shown in Figure 7 and Figure 8, infill percentage and layer thickness greatly influence the tensile properties [24,26]. Layer thickness and width have a significant effect on % elongation values. The contribution of each factor to the total variation is shown in percent (%) in the last column of the tables. According to Table 6, the most influential parameter for the tensile strength is the infill percentage, with a contribution of 67.77%, the layer thickness is second with 11.52%, the layer width is third with 3.88%, and the print speed is last with 0.03%. High infill percentages led to denser internal structures and stronger interlayer bonding, significantly improving tensile strength. On the other hand, layer thickness had the most substantial impact on elongation, as thinner layers contributed to better layer adhesion and ductility. The influence of print speed on both the tensile strength and elongation responses was minimal, suggesting that speed variations do not significantly affect the bonding quality or structural integrity within the tested range.

While the current study focuses on the mechanical performance of neat PLA fabricated via fused deposition modeling (FDM), it is important to contextualize these results within the broader landscape of reinforced PLA composites, which are increasingly used in structural and functional applications due to their enhanced mechanical, thermal, and morphological characteristics. Reinforced PLA systems, such as PLA blended with natural fibers (e.g., flax, hemp, wood flour), glass fibers, or nanoparticles (e.g., carbon nanotubes, graphene, nanoclay), demonstrate significantly improved tensile strength, flexural resistance, and impact behavior compared to neat PLA. These reinforcements contribute to improved stress transfer, reduced brittleness, and enhanced thermal stability, making the composite systems more suitable for load-bearing or high-performance applications [39,40,41,42].

The prediction equations for tensile strength given in Equation (2) were obtained by a regression analysis. A linear regression model was developed to predict tensile strength based on the selected input parameters, with a coefficient of determination (R^2^) of 83.14%, indicating a satisfactory fit. The equation indicated that the fit of the experiment is satisfactory. The regression equation is given below.

(2) Tensile Strength = 18.9 + 0.009 Print speed + 0.3465 Infill percentage − 17.9 Thickness − 10.3 Width

The regression model mentioned above is valid for pure PLA material and only provides information about tensile strength within the given process parameters range.

**Figure 9:**
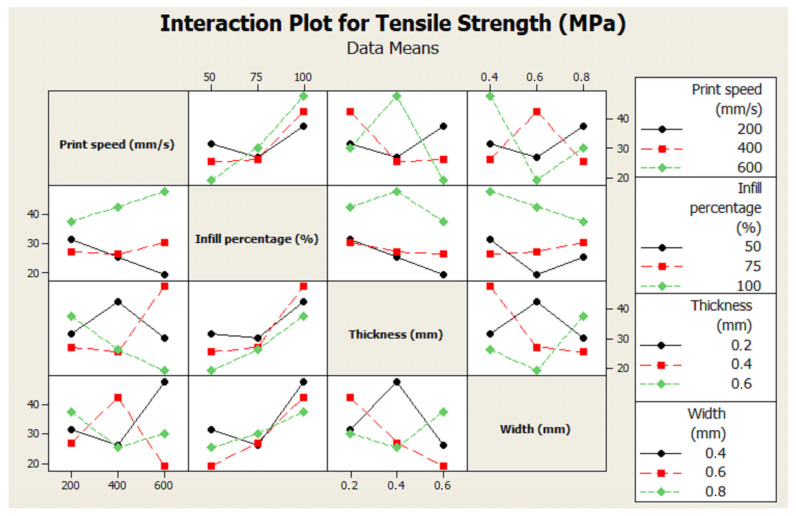
Interaction graph for effects of print speed, infill percentage, layer thickness, and layer width parameters on tensile strength.

**Figure 10:**
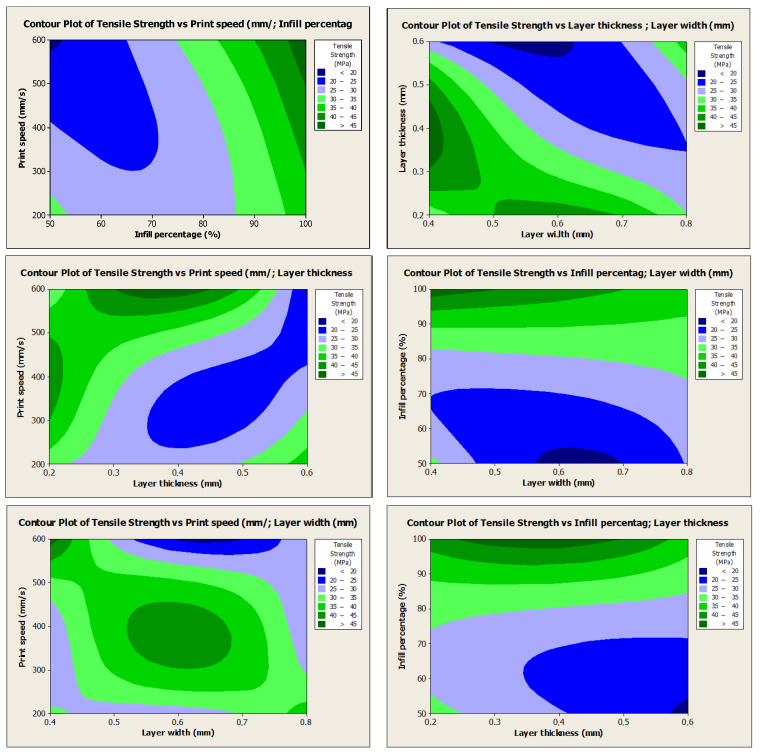
Contour plot for tensile strength.

An interaction plot for tensile strength and contour plot for tensile strength are given in Figure 9 and Figure 10. The interaction plot illustrates the effects and interactions of four key FDM process parameters on the tensile strength of PLA specimens: print speed (200, 400, 600 mm/s), infill percentage (50%, 75%, 100%), layer thickness (0.2, 0.4, 0.6 mm), and layer width (0.4, 0.6, 0.8 mm). The figure presents pairwise interaction plots, with each subplot showing how the mean tensile strength changes across levels of one factor, grouped by levels of another. Higher infill (100%) consistently yields higher tensile strength across all print speeds. At a lower print speed (200 mm/s), strength increases significantly with higher infill, suggesting more complete fusion. The print speed of 600 mm/s tends to reduce tensile strength, especially at lower infill. At 100% infill, thinner layers (0.2 mm) result in higher tensile strength, likely due to better interlayer adhesion. Increasing the thickness to 0.6 mm reduces strength, especially at low infill percentages. At 100% infill, wider extrusions (0.8 mm) yield better tensile strength. However, inconsistent trends at 50% and 75% infill suggest this effect is not linear or may depend on other parameter interactions. At low print speed (200 mm/s), increasing thickness to 0.4 mm slightly improves strength, but beyond that (0.6 mm), it decreases. A high print speed (600 mm/s) consistently lowers tensile strength regardless of thickness, possibly due to insufficient interlayer bonding. A width of 0.6 mm generally yields better tensile strength at a medium print speed (400 mm/s). At high speed (600 mm/s), strength decreases for all widths, indicating a limit to extrusion quality at high speeds. At lower thicknesses (0.2 mm), tensile strength is relatively high for all widths. The combination of large thickness (0.6 mm) and small width (0.4 mm) results in significantly reduced strength, likely due to poor overlap and bonding.

Overall, a higher infill percentage and lower print speed are strongly associated with improved tensile strength. The layer thickness and width show complex interactions, but thinner layers and moderate-to-high widths generally favor higher strength. These findings highlight the importance of optimizing FDM parameters individually and in combination to achieve maximum mechanical performance in PLA-based components.

Figure 10 indicates the influences of the print speed, infill percentage, layer thickness, and layer width parameters on the tensile strength. In terms of tensile strength, a higher tensile strength is obtained at a high infill percentage, high print speed, and low layer thickness and width. Otherwise, low tensile strength, low infill percentage, low printing speed, and high layer thickness and width can be selected.

**Figure 11:**
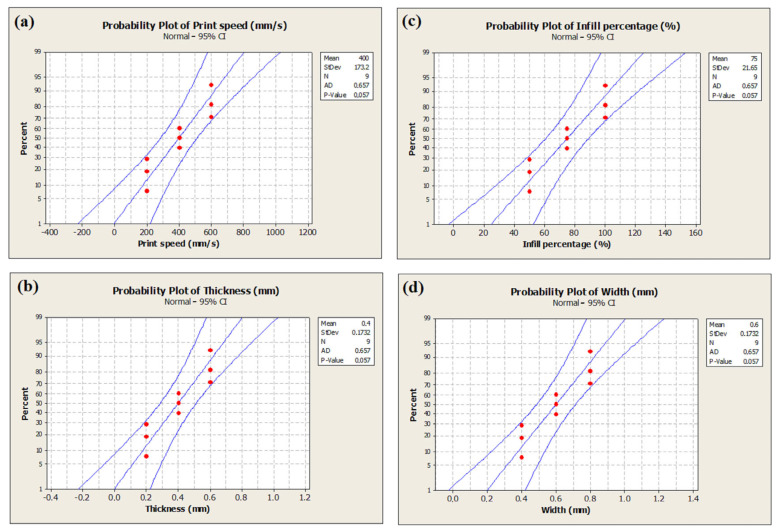
Probability plots: (**a**) print speed; (**b**) infill percentage; (**c**) layer thickness; (**d**) layer width.

The probability plots were utilized to assess how well the experimental data conformed to a theoretical distribution model. The data follow a normal distribution when the predicted values align along a straight line in such plots. As illustrated in Figure 11, the data points aligned closely with the reference line, suggesting a good fit to the normal distribution. Additionally, the *p*-value exceeding 0.05 further supports the normality assumption, affirming the validity of using this data set for statistical modeling and optimization purposes [43].

The outcomes of this study offer valuable contributions to optimizing FDM process parameters aimed at enhancing the mechanical behavior of PLA parts. Through a structured analysis of parameter effects, this research provides actionable recommendations for professionals seeking to improve the tensile performance of FDM-manufactured components. These insights are significant for application areas such as rapid prototyping, biomedical tooling, and end-user products, where mechanical integrity and efficient material usage are essential considerations.

**Figure 12:**
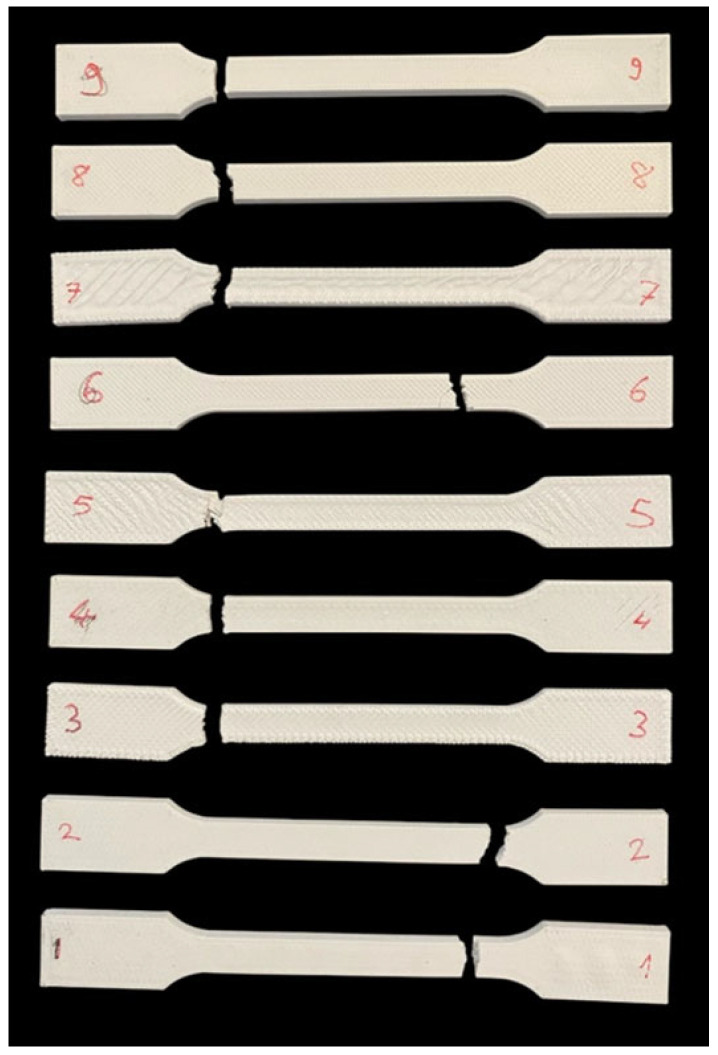
View of one group of test specimens after the tensile test.

**Figure 13:**
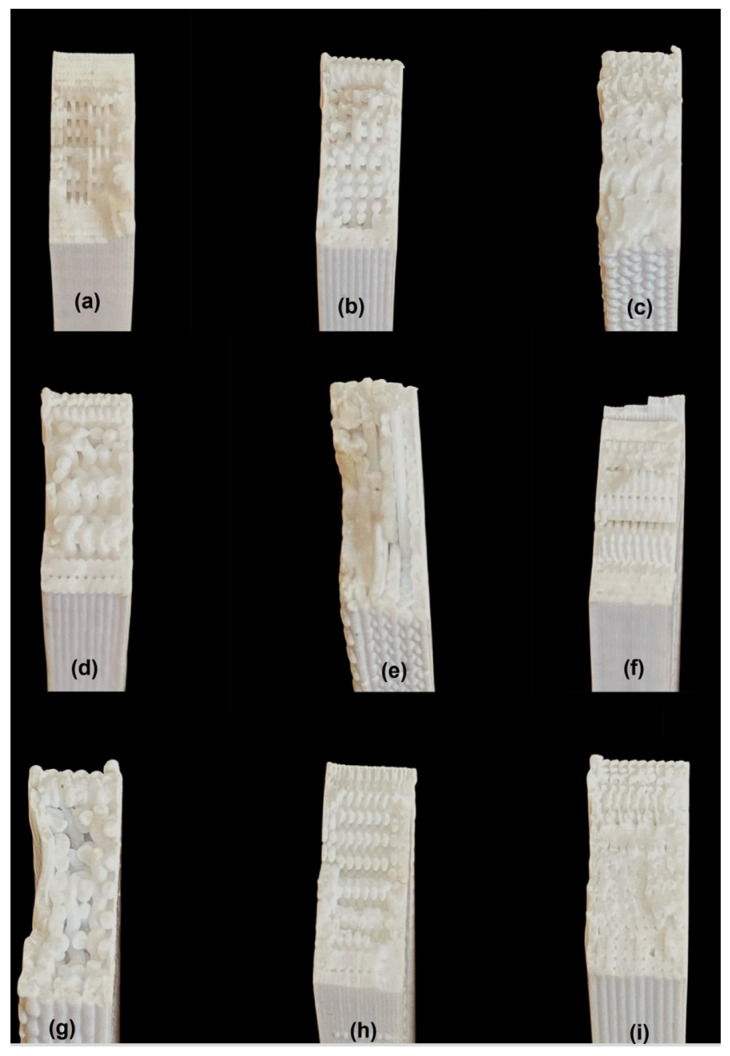
Macro images of the fracture surfaces formed after the tensile test. (**a**) Experiment number 1; (**b**) experiment number 2; (**c**) experiment number 3; (**d**) experiment number 4; (**e**) experiment number 5; (**f**) experiment number 6; (**g**) experiment number 7; (**h**) experiment number 8; (**i**) experiment number 9.

A view of one group of test specimens after the tensile test is given in Figure 12. Macro images of the fracture surfaces formed after the tensile test are shown in Figure 13.

The sample in Figure 13i is experiment number 9 and has the highest tensile strength. This specimen was produced with a print speed of 600 mm/s, an infill percentage of 100%, a layer thickness of 0.4 mm, and a layer width of 0.4 mm. The lowest tensile strength value was obtained in the specimen where the print speed was 600 mm/s, the layer thickness and layer width were 0.6, and the infill percentage was 50% (Figure 13g). The sample with low tensile strength had more irregularities and gaps on its fracture surface. The increase in layer thickness and width caused significant decreases in tensile strength.

Figure 13c,i (experiments 3 and 9) show the most cohesive structures. These use high infill percentages and appropriate combinations of layer thickness and width. Figure 13d,g (experiments 4 and 7) exhibit extreme delamination and brittle fracture, caused by high speed, low infill, and thicker layers that compromise bonding. Figure 13b,h offer relatively balanced results with average infill and print speed. Layer thickness and width remain critical to internal cohesion.

The fracture surface morphology directly correlates with the FDM process parameters. Specimens with high infill percentage, lower print speed, and thinner layers tend to show ductile fractures and better mechanical strength. Conversely, high speed, low infill, and large thickness parameters lead to void formation, layer separation, and brittle failure.

**Figure 14:**
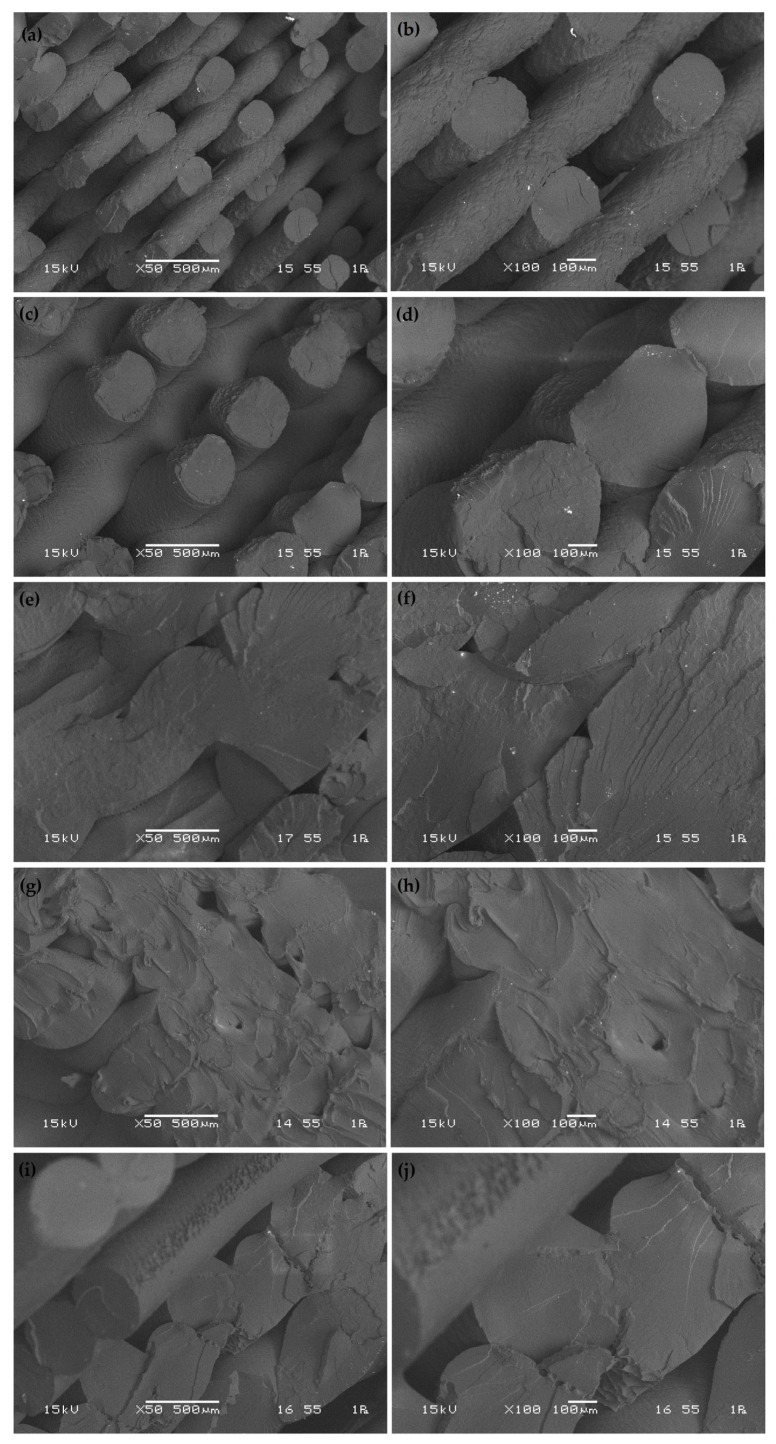
SEM images of the fracture interfaces of the samples: (**a**,**b**) experiment number 1; (**c**,**d**) experiment number 2; (**e**,**f**) experiment number 3; (**g**,**h**) experiment number 4; (**i,j**) experiment number 5.

**Figure 15:**
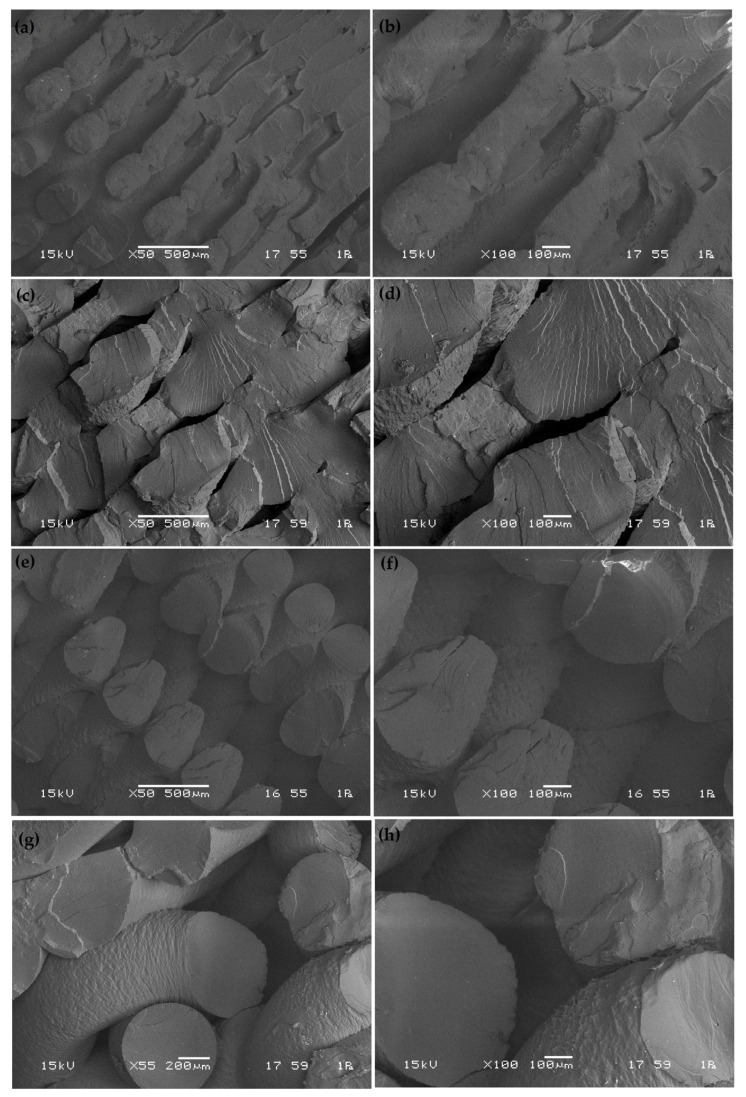
SEM images of the fracture interfaces of the samples: (**a**,**b**) experiment number 6; (**c**,**d**) experiment number 7; (**e**,**f**) experiment number 8; (**g**,**h**) experiment number 9.

To better understand the failure mechanisms associated with different process parameters, a scanning electron microscope (SEM) analysis was conducted on the fracture surfaces of selected PLA samples, as shown in Figure 14 and Figure 15.

In Figure 14, SEM images (a) and (b) (experiment 1 (200 mm/s, 50% infill, 0.2 mm thickness, 0.4 mm width, tensile strength: 31.46 MPa)) display relatively smooth and layered fracture features with moderate interlayer adhesion. The fracture surface indicates a semi-brittle failure with some degree of plastic deformation, corresponding to moderate tensile strength. The presence of visible raster lines suggests limited fusion between adjacent filaments, likely due to the low infill percentage. The moderate strength can be attributed to limited filament fusion due to the low infill rate, which creates discontinuities acting as crack initiation sites. Similar behavior was reported by Chacón et al. [2]. SEM images (c) and (d) (experiment 2 (200 mm/s, 75% infill, 0.4 mm thickness, 0.6 mm width, tensile strength: 27.10 MPa)) show voids and poor interlayer bonding, confirming a brittle fracture mode. The increased layer thickness and wide filament path may have reduced the contact area between deposited layers, decreasing bonding strength and promoting early crack propagation. The combination of thicker layers and increased width hinders thermal consolidation, leading to premature failure—a phenomenon also noted by Domingo-Espin et al. [13]. The fracture surfaces in (e) and (f) (experiment 3 (200 mm/s, 100% infill, 0.6 mm thickness, 0.8 mm width, tensile strength: 37.51 MPa)) exhibit dense deposition with fewer gaps, indicating improved mechanical interlocking and thermal fusion. The structure is more uniform despite the relatively large layer thickness and width, likely benefiting from the full infill support, resulting in higher tensile performance. SEM images (g) and (h) (experiment 4 (400 mm/s, 50% infill, 0.4 mm thickness, 0.8 mm width, tensile strength: 25.37 MPa)) show distinct interfacial gaps and layered separation, with signs of delamination. The combination of a high print speed and low infill negatively affects material consolidation, leading to weak bonding and reduced strength. Fracture surfaces (i) and (j) (experiment 5 (400 mm/s, 75% infill, 0.6 mm thickness, 0.4 mm width, tensile strength: 26.16 MPa)) display partial interlayer contact with some rough texture. Although the narrower layer width improves path precision, the thick layers and moderate infill lead to insufficient bonding, correlating with the observed low tensile strength. In images (a) and (b) of Figure 15 (experiment 6 (400 mm/s, 100% infill, 0.2 mm thickness, 0.6 mm width, tensile strength: 42.56 MPa)), the sample exhibits well-fused layers with minimal porosity, suggesting excellent interlayer adhesion. The small layer thickness ensures consistent heat accumulation, promoting fusion and producing one of the highest tensile strengths among all samples. Previous studies have linked comparable microstructural characteristics to strong ductile performance [6]. SEM images (c) and (d) (experiment 7 (600 mm/s, 50% infill, 0.6 mm thickness, 0.6 mm width, tensile strength: 19.10 MPa)) illustrate severe interlayer voids and brittle fracture morphology, characteristic of weak structural integrity. The fast print speed, thick layers, and low infill limit thermal bonding, drastically reducing tensile performance. Fracture images (e) and (f) (experiment 8 (600 mm/s, 75% infill, 0.2 mm thickness, 0.8 mm width, tensile strength: 30.19 MPa)) show mixed brittle and ductile characteristics, with moderately fused regions. The low layer thickness helps fusion, but the wide paths and high speed still result in inconsistencies across layers. SEM images (g) and (h) (experiment 9 (600 mm/s, 100% infill, 0.4 mm thickness, 0.4 mm width, tensile strength: 47.84 MPa)) demonstrate excellent fusion with uniform and continuous layer structures, indicating optimized interfacial bonding and minimal defects. The fracture surface is relatively smooth and cohesive, consistent with the highest tensile strength among all samples. These features have been reported in prior high-strength FDM-PLA research [44].

## 4. Conclusions

This study systematically investigated the effects of key FDM process parameters, print speed, infill percentage, layer thickness, and layer width, on the tensile properties of 3D-printed PLA specimens using a Taguchi experimental design and statistical analysis. Tensile testing revealed that the highest tensile strength (47.84 MPa) was achieved with the parameter combination of 600 mm/s print speed, 100% infill percentage, 0.4 mm layer thickness, and 0.4 mm layer width. The most influential factor on tensile strength was found to be the infill percentage, contributing 67.77% according to the ANOVA results, while the print speed had the least effect (0.03%). For elongation, layer thickness and layer width were the dominant parameters. Optimization using the S/N ratio identified the ideal process settings for maximizing tensile strength and elongation, and a linear regression model with an R^2^ of 83.14% was successfully developed to predict tensile strength based on the selected input parameters.

Additionally, probability plots confirmed that the experimental data followed a normal distribution, supporting the statistical methods’ reliability. Interaction plots and contour plots for tensile strength showed the effects and interactions of four key FDM process parameters on the tensile strength of PLA specimens. An SEM analysis revealed that samples with high strength showed a more ductile fracture morphology, while samples with low strength showed a brittle fracture morphology.

The findings of this research provide valuable insights into the relationship between FDM parameters and the mechanical performance of PLA parts. They highlight the importance of parameter optimization in enhancing structural integrity, particularly for load-bearing applications. Future work could extend these results by incorporating other materials, environmental effects, or additional mechanical tests, such as fatigue or impact resistance, to broaden the understanding of FDM-printed PLA components. Future work can extend this study by incorporating more advanced materials such as fiber-reinforced PLA composites; analyzing other mechanical properties such as fatigue, impact, or flexural strength; and applying multi-objective optimization techniques to balance strength, ductility, cost, and print time. Additionally, integrating machine learning models could further enhance predictive accuracy and support real-time process control in additive manufacturing.

## Figures and Tables

**Table 1:** Process parameters and their corresponding level values.

Process Parameter	Symbol	Level 1	Level 2	Level 3
Print speed (mm/s)	A	200	400	600
Infill percentage (%)	B	50	75	100
Layer thickness (mm)	C	0.2	0.4	0.6
Layer width (mm)	D	0.4	0.6	0.8

**Table 2:** L9 orthogonal array for the design of experiments.

Experiment Number	Print Speed (mm/s)	Infill Percentage (%)	Layer Thickness (mm)	Layer Width (mm)
1	200	50	0.2	0.4
2	200	75	0.4	0.6
3	200	100	0.6	0.8
4	400	50	0.4	0.8
5	400	75	0.6	0.4
6	400	100	0.2	0.6
7	600	50	0.6	0.6
8	600	75	0.2	0.8
9	600	100	0.4	0.4

**Table 3:** The tensile test results and S/N analysis.

Experiment Number	Tensile Strength (MPa)	S/N of Tensile Strength	% Elongation	S/N of % Elongation
1	31.46	29.9552	0.098	−20.1755
2	27.10	28.6594	0.042	−27.5350
3	37.51	31.4829	0.038	−28.4043
4	25.37	28.0864	0.036	−28.8739
5	26.16	28.3528	0.067	−23.4785
6	42.56	32.5800	0.080	−21.9382
7	19.10	25.6207	0.041	−27.7443
8	30.19	29.5973	0.043	−27.3306
9	47.84	33.5958	0.042	−27.5350

**Table 4:** Response table for the S/N of tensile strength.

Level	Print Speed	Infill Percentage	Layer Thickness	Layer Width
1	30.03	27.89	30.71	30.63
2	29.67	28.87	30.11	28.95
3	29.60	32.55	28.49	29.72
Delta	0.43	4.67	2.23	1.68
Rank	4	1	2	3

**Table 5:** Response table for S/N of % elongation.

Level	Print Speed	Infill Percentage	Layer Thickness	Layer Width
1	−25.37	−25.60	−23.15	−23.73
2	−24.76	−26.11	−27.98	−25.74
3	−27.54	−25.96	−26.54	−28.20
Delta	2.77	0.52	4.83	4.47
Rank	3	4	1	2

**Table 6:** ANOVA table of tensile strength.

Source	DF	SS	MS	F-Value	*p*-Value	Cont %
Print speed	1	0.187	0.187	0.01	0.939	0.03
Infill percentage	1	450.320	450.320	16.07	0.016	67.77
Layer thickness	1	76.612	76.612	2.73	0.174	11.52
Layer width	1	25.585	25.585	0.91	0.393	3.88
Error	4	112.069	28.017			16.80
Total	8	664.773				100

DF: degree of freedom, SS: sum of squares, MS: mean of squares, Cont %: percentage contribution.

## Data Availability

The original contributions presented in the study are included in the article; further inquiries can be directed to the corresponding author.
